# MK-571, a Cysteinyl Leukotriene Receptor 1 Antagonist, Inhibits Hepatitis C Virus Replication

**DOI:** 10.1128/AAC.02078-19

**Published:** 2020-05-21

**Authors:** Isaac Ruiz, Quentin Nevers, Eva Hernández, Nazim Ahnou, Rozenn Brillet, Laurent Softic, Flora Donati, Francois Berry, Sabah Hamadat, Slim Fourati, Jean-Michel Pawlotsky, Abdelhakim Ahmed-Belkacem

**Affiliations:** a Institut Mondor de Recherche Biomédicale (IMRB), INSERM U955, Team “Viruses, Hepatology, Cancers”, Hôpital Henri Mondor, Université Paris-Est, Créteil, France; b Department of Hepatology, Hôpital Henri Mondor, Université Paris-Est, Créteil, France; c National Reference Center for Viral Hepatitis B, C and D, Department of Virology, Hôpital Henri Mondor, Université Paris-Est, Créteil, France

**Keywords:** cysteinyl leukotriene receptor 1, hepatitis C virus, virology

## Abstract

The quinoline MK-571 is the most commonly used inhibitor of multidrug resistance protein-1 (MRP-1) but was originally developed as a cysteinyl leukotriene receptor 1 (CysLTR1) antagonist. While studying the modulatory effect of MRP-1 on anti-hepatitis C virus (HCV) direct-acting antiviral (DAA) efficiency, we observed an unexpected anti-HCV effect of compound MK-571 alone. This anti-HCV activity was characterized in Huh7.5 cells stably harboring a subgenomic genotype 1b replicon. A dose-dependent decrease of HCV RNA levels was observed upon MK-571 administration, with a 50% effective concentration (EC_50_ ± standard deviation) of 9 ± 0.3 μM and a maximum HCV RNA level reduction of approximatively 1 log_10_. MK-571 also reduced the replication of the HCV full-length J6/JFH1 model in a dose-dependent manner. However, probenecid and apigenin homodimer (APN), two specific inhibitors of MRP-1, had no effect on HCV replication. In contrast, the CysLTR1 antagonist SR2640 increased HCV-subgenomic replicon (SGR) RNA levels in a dose-dependent manner, with a maximum increase of 10-fold. In addition, a combination of natural CysLTR1 agonist (LTD4) or antagonists (zafirlukast, cinalukast, and SR2640) with MK-571 completely reversed its antiviral effect, suggesting its anti-HCV activity is related to CysLTR1 rather to MRP-1 inhibition. In conclusion, we showed that MK-571 inhibits HCV replication in hepatoma cell cultures by acting as a CysLTR1 receptor antagonist, thus unraveling a new host-virus interaction in the HCV life cycle.

## Abstract (precis)

The quinoline MK-571 is the most commonly used inhibitor of multidrug resistance protein-1 (MRP-1) but was originally developed as a cysteinyl leukotriene receptor 1 (CysLTR1) antagonist. While studying the modulatory effect of MRP-1 on anti-hepatitis C virus (HCV) direct-acting antiviral (DAA) efficiency, we observed an unexpected anti-HCV effect of compound MK-571 alone. This anti-HCV activity was characterized in Huh7.5 cells stably harboring a subgenomic genotype 1b replicon. A dose-dependent decrease of HCV RNA levels was observed upon MK-571 administration, with a 50% effective concentration (EC_50_ ± standard deviation) of 9 ± 0.

## INTRODUCTION

Hepatitis C virus (HCV) is a blood-borne, single-stranded, positive-sense RNA virus belonging to the *Hepacivirus* genus of the *Flaviviridae* family. During the HCV life cycle, the viral genome of approximatively 9,600 nucleotides is translated into a polyprotein that is subsequently cleaved by cellular and viral proteases into 3 structural proteins (E1, E2, and core) and 7 nonstructural proteins (p7, NS2, NS3, NS4A, NS4B, NS5A, and NS5B) (1). Nonstructural proteins NS3, NS4A, NS4B, NS5A, and NS5B associate with host proteins to form the viral replication machinery, while p7 and NS2 are essential for infectious virus production (2).

Worldwide, 71 million people are estimated to be infected with HCV, representing approximately 1% of the world population, most of whom have chronic liver disease. Chronic HCV infection causes almost 400,000 deaths annually, principally from the complications of cirrhosis or hepatocellular carcinoma (3). Highly efficacious and well-tolerated combinations of direct-acting antiviral (DAA) drugs have revolutionized HCV treatment. Infection cure rates higher than 95% can now be achieved, with a measurable impact on HCV-related morbidity and mortality (4). Four main classes of DAAs are commercially available, including NS3/4A protease inhibitors, NS5A protein inhibitors, nucleoside analogs, and nonnucleoside inhibitors of the NS5B RNA polymerase (5).

Despite the spectacular virological results of current anti-HCV therapies, several issues remain. In patients who fail to achieve a cure of the infection, HCV variants carrying resistance-associated substitutions (RASs) on their genome, i.e., substitutions that confer reduced susceptibility to the administered drugs, are generally selected (6). Their long-term persistence after treatment raises issues as to subsequent retreatment. Although the global rate of treatment failure is low with current DAA combinations, the absolute number of patients requiring retreatment is high. This number will further increase due to the large number of patients who will be treated, in the context of the World Health Organization endeavor to eliminate HCV as a major public health threat by 2030 (3). Importantly, some regions of the world (e.g., central Africa and Southeast Asia) harbor unusual subtypes of known genotypes that are inherently resistant to commonly administered DAAs (7, 8). In addition, the high cost of last-generation DAA regimens limits access to care in low-income areas, while the management of special patient groups, such as those with advanced liver disease or renal failure, may be problematic with current drugs.

Multidrug resistance (MDR), i.e., cell ability to acquire drug resistance, is mainly mediated by the overexpression of membrane drug transporters, such as P‐glycoprotein (P‐gp), breast cancer resistance protein (BCRP), or multidrug resistance protein-1 (MRP-1), which belong to the ATP-binding cassette (ABC) transporter superfamily (9, 10). These transporters influence drug pharmacokinetics, particularly their distribution, thereby modifying their concentrations in cells (11). Drug-drug interactions may occur at the transporter level and modulate drug efficacy and/or toxicity (12). Functional interactions between anti-HCV DAAs and ABC transporters have been reported (4, 13). Indeed, almost all of the approved HCV inhibitors, including sofosbuvir, daclatasvir, ledipasvir, velpatasvir, voxilaprevir, paritaprevir, dasabuvir, glecaprevir, and pibrentasvir, are substrates and/or inhibitors of at least one ABC transporter (4, 14).

To investigate the involvement of ABC transporters in the efflux of HCV protease inhibitors, we had tested the *in vitro* anti-HCV activity of the NS3-4A protease inhibitor telaprevir, alone or in combination with LY335979 (15), KO143 (16), or MK-571 (17, 18), inhibitors of P-gp, BCRP, and MRP-1, respectively. In the control experiments, we observed an unexpected antiviral effect of MK-571 alone, a result that prompted us to characterize the anti-HCV activity of this compound and identify its target.

In addition to MRP-1, MK-571 has been reported to target cysteinyl leukotriene receptor 1 (CysLTR1) (18). Cysteinyl LTs include LTC4, LTD4, and LTE4. They are lipid mediators derived from arachidonic acid (AA) via the 5-lipoxygenase pathway (19, 20). Their biological effects are mediated by distinct CysLTRs belonging to the G protein-coupled receptor family. CysLTRs have been reported to be involved in inflammation, shock, allergic reactions, plasma extravasations, and liver injury (21–23). CysLTR1 has been extensively studied, and selective antagonists have been developed (24–26). They include montelukast (Singulair; Merck, New York, USA) and zafirlukast (Accolate; AstraZeneka, London, UK), which are used for the treatment of bronchial asthma and allergic rhinitis (27).

In the present study, based on our serendipitous discovery of the anti-HCV activity of MK-571, we characterized the antiviral activity of this compound in cellular HCV models and identified its target.

## RESULTS

### MK-571 inhibits HCV replication.

**FIG 1:**
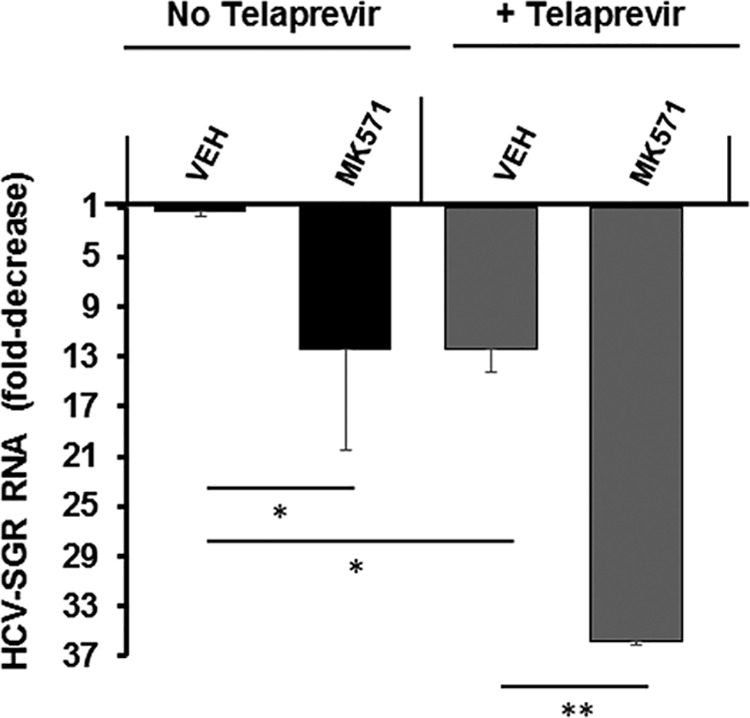
HCV-SGR RNA levels in Huh7.5-SGR cells were quantified by means of RT-qPCR in the absence (black bars) or in the presence (gray bars) of 1 μM telaprevir, without (VEH, vehicle), or in combination with 50 μM MK-571. The data are shown as mean ± SD of at least three independent experiments. *, *P* < 0.05; **, *P* < 0.01.

With the goal to assess the effect of inhibiting ABC transporter (e.g., MRP-1) efflux on the antiviral activity of the HCV protease inhibitor telaprevir, we measured HCV RNA levels in Huh7.5 cells stably harboring a genotype 1b HCV-SGR treated with 1 μM of telaprevir in combination with 50 μM of MK-571. In our control experiments with MK-571 in the absence of telaprevir, a 12-fold decrease of HCV RNA levels was observed, suggesting that MK-571 bears anti-HCV activity. The antiviral effect of 50 μM of MK-571 was equivalent to the effect of 1 μM of telaprevir (Fig. 1). In the presence of telaprevir, MK-571 increased by approximately 3-fold the anti-HCV effect of telaprevir (Fig. 1).

### MK-571 blocks HCV replication in a dose-dependent manner.

**FIG 2:**
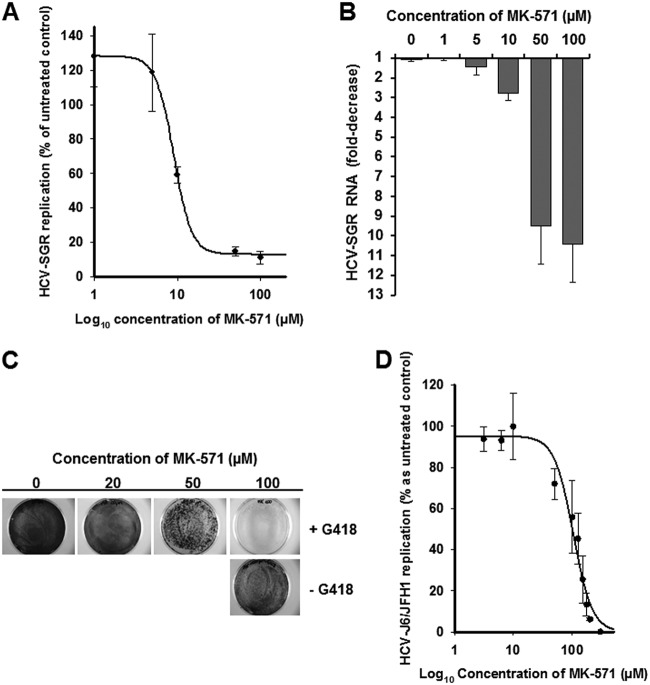
Anti-HCV activity of MK-571. (A) Dose-response curve and (B) fold decrease of MK-571 antiviral activity in Huh7.5 cells stably expressing an HCV genotype 1b subgenomic replicon. Data are shown as means ± SD from at least two independent experiments performed in triplicate. (C) Crystal violet staining of Huh7.5-SGR cells after 2 weeks of treatment with different concentrations of MK-571 in the presence of G418; (D) dose-response curve of MK-571 activity in Huh7.5 cells infected with J6/JFH1 virus strain.

In Huh7.5 cells stably harboring a genotype 1b HCV-SGR, 48 h of treatment with MK-571 induced a dose-dependent decrease of HCV-SGR RNA, with an 50% effective concentration (EC_50_) of 9.0 ± 0.3 μM (Fig. 2A) and a maximal HCV-SGR RNA reduction of approximatively 11-fold at 50 μM (Fig. 2B). Huh7.5 cell viability was measured, and the MK-571 50% cytotoxic concentration (CC_50_) was >100 μM, the highest concentration tested (Table 1).

**TABLE 1:** *In vitro* cellular toxicities of the indicated compound in Huh7.5 cells

Compound	CC_50_ (μM) in Huh7.5 cells
MK-571	147.8 ± 6.8
Cinalukast	>100
SR2640	33.4 ± 3.9
Zafirlukast	>100
Telaprevir	>20

We next examined HCV-SGR clearance during 2 weeks of treatment with MK-571. Huh7.5 cells harboring an HCV-SGR containing the neomycin resistance gene were grown in the presence of increasing concentrations of MK-571 under G418 selective pressure; under such pressure, only cells efficiently replicating the HCV-SGR were able to grow. As shown in Fig. 2C, cell growth was not significantly affected by 100 μM of MK-571 in the absence of G418. In contrast, in the presence of G418, MK-571 inhibited cell growth in a dose-dependent manner, as a result of its inhibition of HCV-SGR replication (Fig. 2C). These results indicate that MK-571 blocks an intracellular step of the HCV life cycle.

The anti-HCV activity of MK-571 was then confirmed in a chimeric genotype 2a/2a HCV full-length infectious model (HCV-J6/JFH1) containing a luciferase reporter gene. Huh7.5 cells were infected with HCV-J6/JFH1 in the presence of an increasing concentration of MK-571, and luciferase activity was quantified 48 h after infection. As with HCV-SGR, MK-571 induced a dose-dependent decrease of HCV-J6/JFH1 RNA replication. However, MK-571 anti-HCV activity appeared higher in the genotype 1b HCV-SGR than the genotype 2a HCV full-length infectious model (Fig. 2D).

### MK-571 anti-HCV activity is related to its CysLTR1 antagonist property rather than MRP-1 inhibition.

**FIG 3:**
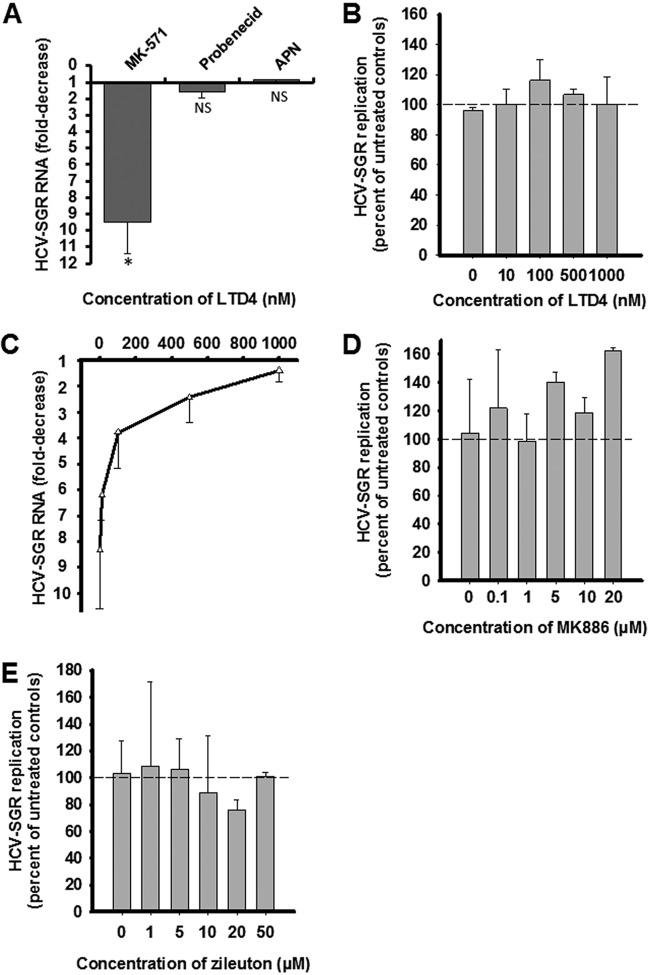
(A) Effect of 50 of μM of MK-571, 1 mM of probenecid, and 0.1 μM of apigenin homodimer (APN) on HCV-SGR RNA levels in Huh7.5-SGR cells. (B) Effect of increasing concentrations of LTD4 on HCV-SGR RNA levels in Huh7.5-SGR cells. (C) Effect of increasing concentrations of LTD4 on the antiviral effect of 50 μM of MK-571 in Huh7.5-SGR cells. (D) Effect of increasing concentrations of MK886 on HCV-SGR RNA levels in Huh7.5-SGR cells. (E) Effect of increasing concentrations of zileuton on HCV-SGR RNA levels in Huh7.5-SGR cells. The data are shown as mean ± SD of at least three independent experiments; NS, not significant; *, *P* < 0.05.

Both MRP-1 and CysLTR1 are known targets of MK-571. To identify which of them is associated with MK-571 anti-HCV activity, we first measured the anti-HCV effect of two other MRP-1 inhibitors, including probenecid (1 mM) (28) and apigenin homodimer (APN; 0.1 μM) (29). As shown in Fig. 3A, none of these two compounds reduced HCV-SGR RNA levels. Furthermore, the addition of 1 mM of probenecid to MK-571 did not modify its anti-HCV activity (data not shown). Together, these results suggest that MRP-1 is not the MK-571 target associated with anti-HCV activity.

Next, the antiviral effect of CysLTR1 natural agonist LTD4 on HCV-SGR was assessed. LTD4 alone did not exert any effect on HCV replication (Fig. 3B). However, increasing concentrations of LTD4 reversed the antiviral effect of 50 μM of MK-571 in a dose-dependent manner, with complete reversion achieved at 1 μM (Fig. 3C).

We examined whether leukotriene biosynthesis is necessary for the HCV life cycle. The antiviral effect of two potent inhibitors of the 5-lypoxygenase (5-LO), MK-886 and zileuton, was assessed. None of them affected HCV-SGR replication (Fig. 3D and E), suggesting that leukotriene biosynthesis is not required for HCV replication.

Together, these results indicate that the anti-HCV activity of MK-571 is related to CysLTR1 and that it can be reverted by the addition of the CysLTR1 natural ligand.

### Anti-HCV activity of CysLTR1 antagonists zafirlukast, cinalukast, and SR2640 and their interaction with MK-571 antiviral activity.

**FIG 4:**
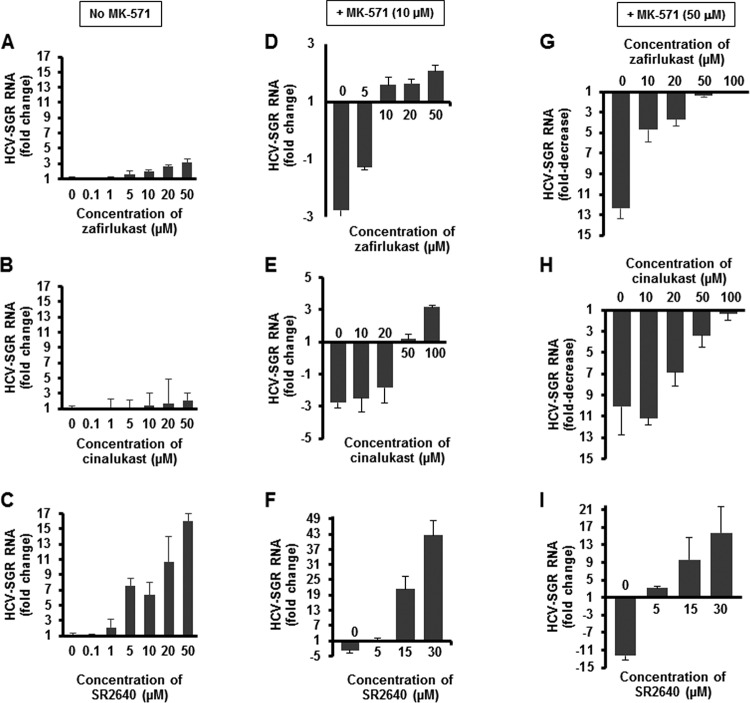
Effect of zafirlukast (A), cinalukast (B), and SR2640 (C) on HCV-SGR RNA expression in Huh7.5-SGR cells in the absence of MK-571. Effect of zafirlukast (D), cinalukast (E), and SR2640 (F) on HCV-SGR RNA expression in Huh7.5-SGR cells in the presence of 10 μM of MK-571. Effect of zafirlukast (G), cinalukast (H), and SR2640 (I) on HCV-SGR RNA expression in Huh7.5-SGR cells in the presence of 50 μM MK-571.

The effect of increasing concentrations of CysLTR1 antagonists zafirlukast, cinalukast, and SR2640 was tested in the absence or in the presence of 50 μM MK-571 in Huh7.5 HCV-SGR cells. As shown in Fig. 4A and B, respectively, zafirlukast and cinalukast alone had a modest proviral effect on HCV RNA replication. SR2640 alone increased HCV-SGR RNA levels in a dose-dependent manner, with a maximum increase of 11-fold achieved at a concentration of 20 μM (Fig. 4C).

Both zafirlukast and cinalukast reversed the anti-HCV activity of MK-571 in a dose-dependent manner, with full reversion achieved at the highest dose tested (Fig. 4D, G, E, and H). The addition of SR2640 to MK-571 not only reversed its anti-HCV activity but also increased HCV replication at the highest concentrations tested (Fig. 4F and I). Together, these results suggest that CysLTR1 regulates HCV replication, in a positive or negative way depending on the CysLTR1 antagonist used.

## DISCUSSION

The approval of highly efficient, well-tolerated DAAs has profoundly changed the HCV treatment landscape. HCV infection can now be cured in most DAA-treated patients within 8 to 12 weeks (4). Despite this unprecedented therapeutic revolution, HCV remains an interesting model to better understand RNA virus life cycles and their interactions with infected hosts.

While studying the modulatory effect of ABC transporter inhibition on the anti-HCV activity of first-generation HCV protease inhibitors, we fortuitously observed an inhibitory effect of MK-571 on HCV replication in a hepatoma cell line harboring an HCV replicon. We further characterized the anti-HCV activity of MK-571 in Huh7.5 cells stably harboring a genotype 1b subgenomic replicon and observed a dose-dependent effect with an EC_50_ of 9.0 ± 0.3 μM and a maximum HCV RNA level reduction of approximatively 11-fold at a concentration of 50 μM of MK-571. Extended treatment with 100 μM of MK-571 cured almost all Huh7.5-SGR-containing cells from their replicons. These results suggest that MK-571 inhibits HCV RNA replication. Although the MK-571 antiviral effect was relatively modest, it points out a new, thus far unknown mechanism involving an interaction between HCV and a host cellular pathway that influences its replication.

The lack of anti-HCV activity of APN and probenecid, two specific MRP-1 inhibitors, and their lack of effect on MK-571 antiviral activity suggested that another target of MK-571 is involved in the interaction with the HCV life cycle. CysLTR1 has been described as another binding target for MK-571 (18). CysLTR1 belongs to the G protein-coupled receptor (GPCR) family. Its activation is linked to the metabolism of phosphatidylinositol and intracellular calcium mobilization (26). CysLTR1 has been shown to activate mitogen-activated protein (MAP) kinases to induce cellular differentiation and proliferation, chemotaxis, and actin reorganization to release mediators of inflammation and to regulate hematopoietic stem cells (25). CysLTR1 has been implicated in a number of inflammatory diseases, including asthma and allergic rhinitis (21, 23). Several CysLTR1-specific antagonists, including montelukast, cinalukast, and zafirlukast, have been developed and are used in clinical practice for the treatment of these diseases (30). Recent studies suggested that CysLTR1 is also involved in various types of liver diseases (31). The human CysLTR1 gene encodes a 337-amino acid protein with a calculated molecular mass of 38 kDa, reported to migrate at a molecular weight of approximately 44 kDa in its monomeric form, which contains a nuclear localization signal at its C-terminal end (25).

Our results demonstrate that the antiviral activity of MK-571 is related to the CysLTR1. Indeed, LTD4, the natural agonist of CysLTR1, reversed the anti-HCV effect of MK-571 in a dose-dependent manner, suggesting that MK-571 displacement from CysLTR1 is sufficient to lose anti-HCV activity. Unlike MK-571, LTD4 and 5-LO inhibitors alone had no effect on HCV replication, suggesting that the presence of CysLTR1 rather than its ligand-induced activation is exploited by HCV for its replication. Interestingly, none of the other CysLTR1 antagonists tested, including zafirlukast, cinalukast, and SR2640, reduced HCV RNA replication; however, all of them reversed the anti-HCV activity of MK-571. SR2640 induced HCV replication in a dose-dependent manner, suggesting that CysLTR1 has an effect on the HCV life cycle that is not univocal, according to the compound and its binding mode. This differential effect could be explained by the fact that receptor binders may act as partial agonists, neutral agonists, or inverse agonists (32, 33), thereby inducing different biological responses.

In summary, we serendipitously identified MK-571 as an inhibitor of HCV replication in hepatoma cell lines harboring an HCV replicon. MK-571 anti-HCV activity was not related to its well-known effect as an MRP-1 inhibitor but rather to its CysLTR1 antagonist property. Our results highlight, for the first time, the implication of the CysLTR1 in the HCV life cycle. Overall, our study demonstrates that CysLTR1 is involved in HCV replication, with an impact on both the HCV life cycle and infected cell biology.

## MATERIALS AND METHODS

### Drugs.

MK-571, MK-886, zileuton, probenecid, cinalukast, and zafirlukast were purchased from Sigma-Aldrich (Saint Louis, MO, USA), while telaprevir was purchased from AGV discovery (Montpellier, France). APN was kindly provided by Attilio Di Pietro (Institut de Biochimie et Chimie des Protéines, Lyon, France). SR2640 was purchased from Tocris (Bristol, UK).

### HCV plasmid.

Plasmid I389-Neo/NS3-3′/5.1, which contains a neomycin resistance gene and an HCV genotype 1b subgenome, was kindly provided by Ralf Bartenschlager (University of Heidelberg, Heidelberg, Germany). This plasmid was transfected into Huh7.5 cells to generate Huh7.5-SGR cells stably harboring an HCV genotype 1b subgenomic replicon (SGR).

### Cell culture.

Human hepatoma-derived Huh7.5 cells (Apath LLC) were cultured in complete Dulbecco’s modified Eagle medium (DMEM; ThermoFisher Scientific, Waltham, MA, USA) supplemented with 10% fetal bovine serum, 50 IU/ml penicillin, 100 μg/ml streptomycin, and 0.1 μg/ml amphotericin B (ThermoFisher Scientific). Huh7.5-SGR cells were cultured in the same medium supplemented with 0.5 mg/ml of G418 (ThermoFisher Scientific) to eliminate Huh7.5 cells that do not harbor the subgenomic replicon.

### Assessment of anti-HCV activity in the subgenomic replicon model.

Huh7.5 cells stably harboring an HCV genotype 1b bicistronic replicon (*I389-Neo/NS3-3′/5.1*) were seeded at the density of 5,000 cells/well in a 96-well plate. The cells were treated for 48 h with the tested compounds diluted in complete DMEM containing 1% dimethyl sulfoxide (DMSO) with 0.5 mg/ml of G418. Total RNA was extracted using the RNeasy 96 kit (Qiagen, Hilden, Germany). HCV-SGR RNA levels were measured by reverse transcriptase quantitative PCR (RT-qPCR) using the TaqMan technology with HCV-specific primers (forward primer, 5′-CGC CCA AAC CAG AAT ACG A-3′; and reverse primer, 5′-AGA TAG TAC ACC CTT TTG CCA GAT G-3′) and a qPCR probe (5′-6-carboxyfluorescein [FAM]-CAA TGT GTC AGT CGG-6-carboxytetramethylrhodamine [TAMRA]-0.3′). The data were analyzed with the 2^−ΔΔ^*^CT^* method, with all samples normalized to glyceraldehyde-3-phosphate dehydrogenase (GAPDH) mRNA. All experiments were performed in triplicate. HCV-SGR RNA relative quantities were plotted against compound concentrations and fitted with a four-parameter logistic curve with SigmaPlot v11 software. EC_50_s were determined from the curves.

### Assessment of antiviral activity in the HCV infectious model.

Huh7.5 cells were incubated 24 h before infection. Then, cells were infected with HCV (J6/JFH1 strain) at an mutiplicity of infection (MOI) of 0.2 during 8 h in the presence of drugs. HCV-infected cells were washed with phosphate-buffered saline (PBS) and incubated for 48 h in fresh medium containing the drugs. Then, luciferase activity was measured and plotted against compound concentrations.

### Cytotoxicity assay.

Assessment of the compound cytotoxicity was performed with the CellTiter 96 AQueous One assay (Promega), according to the manufacturer’s instructions. Huh7.5 cells were seeded at a density of 5,000 cells/well in a 96-well plate and incubated 24 h before addition of increasing concentrations of the indicated compounds. After 48 h, cells were incubated 4 h in the presence of the CellTiter 96 AQueous One solution reagent, and absorbance was measured at 490 nm.

### Statistical analysis.

Statistical analyses were performed using SigmaPlot software. Statistics were calculated using *t* test analysis of variance. *P* values below 0.05 were considered statistically significant.
